# The Effect of Statin Therapy on Inflammatory Biomarkers: A Systematic Review

**DOI:** 10.7759/cureus.18273

**Published:** 2021-09-25

**Authors:** Matthew C Proute, Nageshwar Kothur, Petros Georgiou, Tatsiana Serhiyenia, Wangpan Shi, Mina E Kerolos, Roshini Pradeep, Aqsa Akram, Safeera Khan

**Affiliations:** 1 Internal Medicine, California Institute of Behavioral Neurosciences & Psychology, Fairfield, USA; 2 Research, Oxford University Hospitals NHS Foundation Trust, Oxford, GBR; 3 Pathology, Chulalongkorn University, Bangkok, THA; 4 Pathology, California Institute of Behavioral Neurosciences and Psychology, Fairfield, USA; 5 General Medicine, California Institute of Behavioral Neurosciences & Psychology, Fairfield, USA; 6 Internal Medicine, Dallah Hospital, Riyadh, SAU

**Keywords:** hmg-coa reductase inhibitors, high-sensitivity c-reactive protein, erythrocyte sedimentation rate, inflammatory biomarkers, statin therapy, atorvastatin, simvastatin, lovastatin, pravastatin, pitavastatin

## Abstract

3-hydroxy-3-methylglutaryl-CoA (HMG-CoA) reductase inhibitors are commonly used drugs in the management of elevated lipid levels and cardiovascular disease. In cardiovascular diseases, among other common chronic conditions, inflammatory biomarkers are used to monitor disease progression and the risk of recurrent adverse events. We explored whether or not there was a positive effect on these biomarkers using HMG-CoA reductase inhibitors. The systematic review was conducted by gathering relevant papers mainly from three databases, identified through a generated Medical Subject Headings (MeSH) strategy. Identification of papers was subsequently followed by applying a selected inclusion and exclusion criteria to narrow the papers chosen for review. Post the application of stipulated criteria, 12 papers remained. They were subsequently assessed for risk of bias using a Cochrane risk analysis tool, identifying most as having some concerns of bias or low risk of bias. We found that HMG-CoA reductase inhibitors exhibit both a lipid-lowering effect addition to an anti-inflammatory effect.

## Introduction and background

3-hydroxy-3-methylglutaryl-CoA (HMG-CoA) reductase inhibitors (statins) are the mainstay of management in patients who present with an elevated total cholesterol level, particularly in those with an elevated low-density lipoprotein cholesterol (LDL-C) [1]. Complications of cardiovascular disease are the most common cause of death worldwide, and as such, statin therapy forms an important part of mortality reduction [2,3]. As an extension of their lipid-lowering properties, statins have shown marked efficacy in the reduction of the development of atherosclerotic cardiovascular disease, first-time major adverse cardiovascular events (MACE), as well as the rate of MACE [4]. This class of drugs which includes atorvastatin, fluvastatin, lovastatin, pitavastatin, pravastatin, and rosuvastatin, may be administered as low, moderate, or high intensity and show varying effects on inflammatory markers as well as improved cardiovascular outcomes.

High-sensitivity C-reactive protein (Hs-CRP) and erythrocyte sedimentation rate (ESR) levels are two largely predominant inflammatory biomarkers tested to guide therapy [5]. Although non-specific, they aid in the monitoring of disease progression. Studies suggest that statins provide anti-inflammatory effects by disrupting the relationship between atherosclerotic disease and chronic inflammation [6]. It also raises the question of initiating statin therapy in those deemed to be at high risk of atherosclerotic cardiovascular disease, irrespective of low-density lipoprotein (LDL) levels. Patients with chronic inflammatory conditions such as rheumatoid arthritis have also been shown to benefit from statin intervention through modulation of inflammatory pathways [7].

Although statin therapy has become a mainstay of management in cardiovascular disease through its lipid-lowering effects, the anti-inflammatory properties are still yet to be fully explored and remain inconsistent [1]. This raises the question of whether there is a proven benefit in adding statins as an anti-inflammatory agent in those with chronic inflammatory diseases. As such, this systematic review will assess the anti-inflammatory benefits of statin therapy.

## Review

Method

This systematic review was conducted with the guidance of and strict adherence to the Preferred Reporting Items for Systematic Reviews and Meta-Analyses (PRISMA) guidelines, 2020 [8]. The question generated served to identify if statins exhibited a significant anti-inflammatory effect.

Search Strategy

Two major databases were utilized to obtain our research articles namely PubMed, which by extension encompasses articles in both PubMed Central (PMC) and MEDLINE (Medical Literature Analysis and Retrieval System Online). Science Direct has also been utilized to increase the yield.

To identify the required articles, we generated a Medical Subject Headings (MeSH) strategy using specific keywords and booleans. These included: Inflammatory Biomarkers, Erythrocyte Sedimentation Rate (ESR), High Sensitivity C-Reactive Protein (Hs-CRP), Statin Therapy, HMG-CoA Reductase Inhibitors, atorvastatin, fluvastatin, lovastatin, pitavastatin, pravastatin, rosuvastatin, and simvastatin. The generated MeSH strategy was then plugged into the PubMed search engine yielding articles relating to, but not limited to, the MeSH strategy. Articles were then identified and screened based on titles and available abstracts. Duplicates were further identified and removed from our pool of articles. Selected articles were then further screened using our stipulated inclusion/exclusion criteria and assessed for quality before being chosen for analysis.

Inclusion and Exclusion Criteria

Articles selected for review were limited to randomized clinical trials (RCTs) only to maintain the clinical relevance of the systematic review. These articles encompassed papers that a) were published over 11 years (2010to 2021), b) had patient populations over the age of 18, and c) those published in English Language only.

Papers were excluded if a) full-text articles were unavailable, b) were grey literature, c) they did not directly correlate to the research topic in question.

Assessment of Study Quality

After applying our inclusion/exclusion criteria, all remaining articles were checked for quality using the Cochrane risk-of-bias assessment tool (RoB 2). RoB 2 allowed for the risk of bias in each paper to be stratified based on high, low, or some risk concerns.

Results & analysis

**Figure 1:**
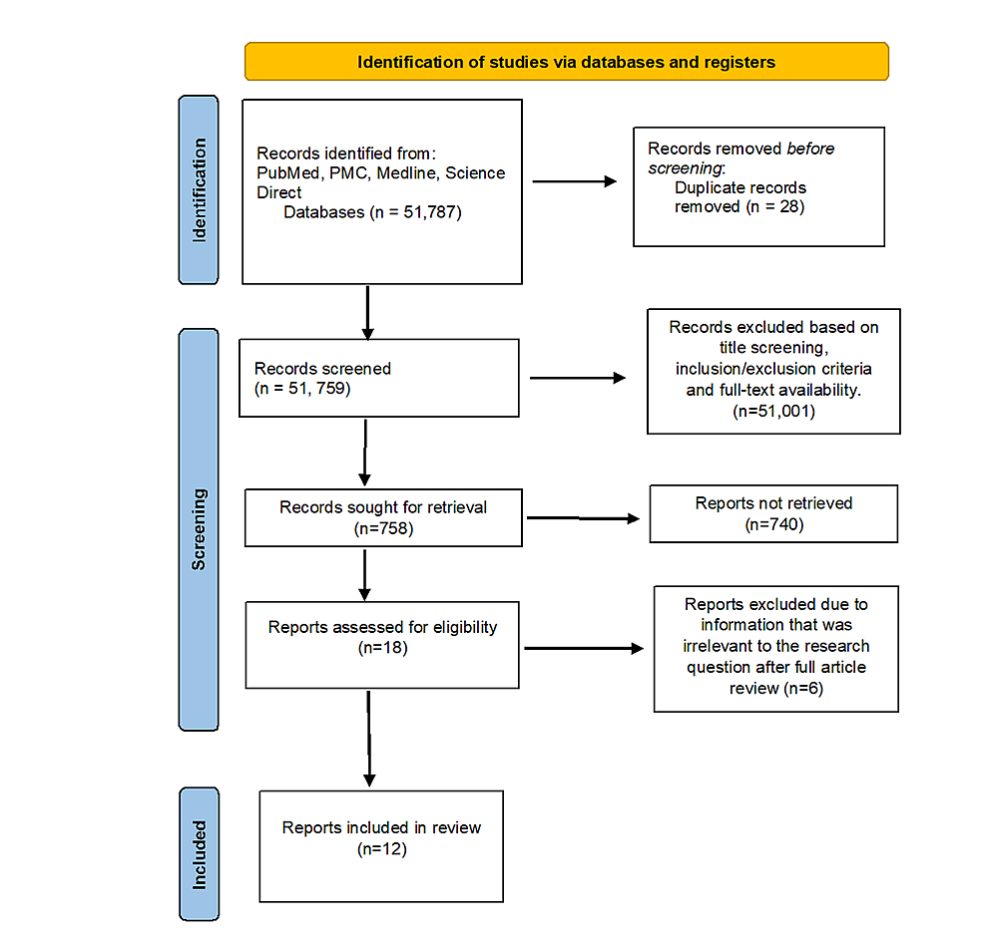
PRISMA flow diagram summarizing identification, screening, and inclusion process PRISMA: Preferred Reporting Items for Systematic Reviews and Meta-Analyses

Our designed MeSh strategy generated a total of 51,787 articles, which we then screened for duplicates and applied the stipulated inclusion and exclusion criteria. This yielded 758 articles out of which 18 were retrieved. Of these 18 articles, six were omitted due to information that was not deemed entirely relevant to the research question. The article selection process is depicted in Figure 1 by the PRISMA flow diagram.

The 12 published papers remaining were selected, and quality checked as stipulated above. Of the 12 papers selected, all were reviewed for this study. Fifty percent were found to have some concerns of bias, 42% were found to have low concerns of bias, with one remaining study being deemed high risk. Table 1 below summarizes the results of the quality assessment. All studies were reviewed despite having some concerns, as evidence remained significant, and most only had one domain showing some concerns.

**Table 1:** Summary of quality assessment using RoB 2 RoB 2: Cochrane risk-of-bias assessment tool

Study		Domain 1		Domain 2A		Domain 2B		Domain 3		Domain 4	Domain 5	Overall Risk of Bias
Barale, C [6]		Some Concerns		Low		Low		Low		Low	Low	Some Concerns
Kawada-Watanabe, E [9]		Low		Low		Some Concerns		Low		Low	Low	Some Concerns
Fang, M [10]		Low		Low		Low		Low		Low	Low	Low
Kitas, GD [11]		Low		Low		Low		Low		Low	Low	Low
Zhao, Y [12]		Low		Low		Some Concerns		Low		Low	Low	Some Concerns
Fuentes-Orozco, C [13]		Low		Low		Low		Low		Low	Low	Low
Bedi, P [14]		Low		Low		Low		Low		Low	Low	Low
Aranow, C [15]	Low		Low		Low		Low		Low		Low	Low
Wu, NQ [16]		Low		Some Concerns		Some Concerns		Low		Low	Some Concerns	Some Concerns
Flannagan, KS [17]		Low		Some Concerns		Low		High Risk		Low	Low	High Risk
Taguchi, I [18]		Low		Low		Some Concerns		Low		Low	Low	Some Concerns
Kitagawa, K [19]		Low		Low		Some Concerns		Low		Low	Low	Some Concerns

As per the inclusion/exclusion criteria, all articles were randomized clinical trials, with a few utilizing the double-blinding method. The vast majority of studies conducted indicate a positive effect on inflammatory markers which we further analyzed.

Discussion

We conducted a thorough analysis of the 12 published research articles that were based on RCTs, to come to an educated conclusion of whether drug therapy with differing statins will lead to an anti-inflammatory effect in addition to its positive effect on lipid reduction and by extension cardiovascular risk.

According to the 2019 American College of Cardiology/American Heart Association (ACC/AHA) guidelines on the primary prevention of cardiovascular disease, patients with an assessed increased risk of atherosclerotic cardiovascular disease (ASCVD) should be considered candidates to commence statin therapy at either moderate or high-intensity dosages [20]. Of the seven statins available, only two qualify for high-intensity therapy i.e., rosuvastatin (40mg/20mg) and atorvastatin (80mg/40mg) [21]. The remaining statins at their highest doses are considered at best moderate-intensity statins [21].

Evidence suggests that elevated Hs-CRP levels in those with chronic inflammatory disease and ASCVD, including acute coronary syndrome (ACS), indicate a much higher mortality rate and increased risk of recurrent cardiovascular events [9]. Although LDL-C levels are an important biochemical marker in monitoring those at risk for disease recurrence/progression, the inflammatory nature of atherosclerosis brings forth the thought that Hs-CRP should also be used as a therapeutic target for these patients [9,10]. Fang et al. found that using Hs-CRP in conjunction with LDL-C levels for monitoring disease progression led to a significant reduction in recurrent adverse cardiac events [10]. This was further justified by Kawada et al. who while analyzing a previous trial conducted on 1734 patients with ACS, found that increased levels of Hs-CRP were directly correlated to adverse cardiac events regardless of the LDL-C levels [9].

High-Intensity Therapy

Kitas et al. investigated a subset of patients (n=3002) who had a chronic inflammatory condition - rheumatoid arthritis (RA) - by randomly subjecting patients to atorvastatin 40mg therapy vs. a placebo therapy [11]. It was discovered that in addition to the significantly lowered LDL-C levels in the study population on atorvastatin, Hs-CRP levels were also lowered considerably by approximately 1mg/L (P<0.0001) in comparison to their placebo group. Although a significant difference, it was indicated that there was no clinical significance in the context of RA [11]. In 2017, Zhao et al. conducted a similar study. Though of a much smaller size (n= 143), the study utilized atorvastatin as its statin of choice at differing doses of 10mg, 20mg, and 40mg. Atorvastatin at 10mg, however, was combined with traditional Chinese medicine of their choice and investigated. As expected, blood lipid levels were significantly lower across all three groups than their baseline levels before commencing therapy. In addition to the lipid-lowering effects, there was a significant lowering of Hs-CRP levels across all three subsets of treatment. However, minimal difference in change between the groups leads us to assume the anti-inflammatory effect may not be dose-dependent [12]. A limiting factor of Zhao et al.'s study, however, in identifying dose-related effects was attributed to the much shorter duration of the study i.e. eight weeks compared to the study by Kitas et al. that lasted approximately two and a half years. This leaves room for the possibility that a longer duration of therapy may result in a greater reduction in inflammatory biomarkers.

Statins, while exhibiting an anti-inflammatory effect, also aid clinically in an immunomodulatory effect. An RCT conducted by Orozco et al. showed a significant (P=0.007) reduction in pre-transplant and post-transplant Hs-CRP in renal transplant donors and recipients [13]. Compared to the larger study by Kitas et al., a similar dose of atorvastatin 40mg vs. placebo therapy was used, although for a much shorter duration of four weeks [11,13]. A pilot RCT in a subset of patients with severe bronchiectasis due to chronic Pseudomonas aeruginosa was also conducted over six months, to looking at the effect of atorvastatin on systemic inflammation and quality of life [14]. In the 32 patient studies (27 completed), it was seen that atorvastatin 80mg administered daily over six months had a significant change in multiple inflammatory markers, including serum C-X-C motif ligand 8 (CXCL8), tumor necrosis factor (TNF), and intercellular adhesion molecule (ICAM). A reduction in Hs-CRP was also noted, however not of statistical significance, with no change in ESR [14]. This contradicts previous findings discussed above by Kitas et al. and Zhao et. al.

In comparison to Kitas et al.’s study, the clinical trial carried out by Aranow et al. in patients with active RA investigated the effect of daily lovastatin 80mg therapy. A much smaller sample size (n=55) with a study duration of 84 days yielded results indicating no significant change in Hs-CRP levels between subjects on lovastatin vs. those on placebo [15]. Lovastatin at a maximum dosage of 80mg, however, is not entirely comparable with atorvastatin at 40mg as lovastatin at best is considered moderate intensity. Aranow et al. also indicated limitations of analysis attributed to the small sample and short duration of follow-up [15].

Dual Lipid-Lowering Therapy

The lowering of lipids in the body can also be done through the use of monotherapy or polytherapy. Those with mixed dyslipidemia may benefit from polytherapy from the addition of drugs such as ezetimibe, bile acid sequestrants, proprotein convertase subtilisin/kexin type 9 (PCSK9) inhibitors, or adenosine triphosphate-citrate lyase inhibitors. 98 patients with ASCVD naïve to lipid-lowering therapy were followed by Wu et al., comparing the use of ezetimibe with moderate-intensity atorvastatin therapy vs. high-intensity atorvastatin therapy only. The study, although primarily observing the lipid-lowering effects of each therapy, also looked at the effect on inflammatory biomarkers in the study population. At weeks four and 12 of therapy, measurements showed some degree of reduction in Hs-CRP levels with continuing reduction at week 12 compared to week four of therapy. There was also a greater reduction in Hs-CRP seen in the high-intensity atorvastatin group when compared with those on combination therapy [16]. Although an anti-inflammatory effect was seen, it was not statistically significant (p>0.5) [16]. Compared with findings from Kitas et al. and Zhao et al.’s studies, these findings indicate anti-inflammatory effects with atorvastatin in general, both with moderate and high intensity, and also indicating a cumulative effect with a duration of therapy [11,12].

It is important to mention that many patients undergoing lipid-lowering therapy with statins are very likely to be on other ASCVD protective agents such as aspirin, clopidogrel, and other therapies outside of alternative lipid-lowering agents. Flannagan et al. investigated the relationship between aspirin 162mg and pravastatin 40mg therapies combined or used singly [17]. They followed a subset of 25 women (n=25) randomized to either therapy or a combination of both equally and checked Hs-CRP at the end of two, three, and four weeks. They had positive reductions in Hs-CRP levels at two weeks in all test groups. However, those on aspirin only had a return to baseline levels as time progressed. In contrast, those on dual therapy or pravastatin therapy only continued to reduce their Hs-CRP levels [17]. Although the study shows supporting evidence that there is an anti-inflammatory effect of pravastatin, the sample size was small compared to other similar studies with a very short follow-up time.

Moderate & Low-Intensity Therapy

A large randomized trial by Taguchi et al. published in 2018, utilizing pitavastatin 4mg per day (moderate-intensity) and pitavastatin 1mg per day (low-intensity), was conducted in an adult Japanese population of median age 68 years. Pitavastatin at both doses showed positive results in reducing LDL-C levels as expected; however, it also showed reductions in Hs-CRP levels in both treatment groups. A significant difference, however, was noted in the Hs-CRP response in the 4mg therapy group with a significant reduction in all-cause mortality [18]. Taguchi et al. also had a greater than 80% follow-up rate throughout the study, with the median time being 3.9 years, which compares well with Kitas et al. [11,18]. Although the study presented important limitations, including an open-label protocol and premature termination of the study, the results still indicated a vast difference in the effects of moderate vs. high-intensity statin therapy, in keeping with comparative studies [18]. Taking a look at simvastatin therapy at a maximum dosage of 40mg daily, 45 patients were screened, selected, and randomly divided into two groups: simvastatin therapy vs. diet only. While Barale et al. did not use the inflammatory markers of ESR and Hs-CRP, they did, however, discover statistically significant improvements in circulating inflammatory markers after just two months of treatment in those on simvastatin therapy as compared with diet only [6].

Data collected does significantly confirm the anti-inflammatory effects of statin therapy, more so in high-intensity therapy. However, limited data on the use of other high-intensity therapies such as rosuvastatin leaves room for interpretation of the efficacy of both rosuvastatin and atorvastatin or the latter alone. The majority of studies analyzed were also of short duration. Statins usually are used as a long-term therapy. As such, the efficacy of a statin as an anti-inflammatory drug would be better understood over a longer duration, especially with lower intensity statins.

## Conclusions

Statins have been shown to exhibit a positive effect on lipid levels in addition to an anti-inflammatory effect. High-intensity statins, in particular atorvastatin, have demonstrated good anti-inflammatory effects with a reduction in adverse cardiac events. Although limited evidence was found as to the efficacy of rosuvastatin, the benefits are still expected to be seen as with atorvastatin. Low and moderate-intensity statins also show significant reductions in inflammatory biomarkers. It has also been seen that dual anti-lipid therapy, in addition to other anti-inflammatory agents combined with statin therapy, provides a significant reduction in systemic inflammation. It is important to continue exploring the use of statins as a mainstay of management, not only for their anti-lipid benefits but also for anti-inflammatory benefits through long-term therapy.
